# Occurrence and characteristics of extended-spectrum β-lactamases producing *Escherichia coli* in foods of animal origin and human clinical samples in Chhattisgarh, India

**DOI:** 10.14202/vetworld.2016.996-1000

**Published:** 2016-09-21

**Authors:** Bhoomika, Sanjay Shakya, Anil Patyal, Nitin Eknath Gade

**Affiliations:** 1 Department of Veterinary Public Health and Epidemiology, College of Veterinary Science and Animal Husbandry, Chhattisgarh Kamdhenu Vishwa Vidyalaya, Anjora, Durg, Chhattisgarh, India; 2 Department of Veterinary Physiology and Biochemistry, College of Veterinary Science and Animal Husbandry, Chhattisgarh Kamdhenu Vishwa Vidyalaya, Anjora, Durg, Chhattisgarh, India

**Keywords:** *bla*_TEM_, *bla*_SHV_, *bla*_CTX-M_, *Escherichia coli*, extended-spectrum β-lactamases

## Abstract

**Aim:**

To assess the prevalence of antimicrobial resistance producing extended-spectrum β-lactamases (ESBL) (*bla*_TEM_, *bla*_SHV_, and *bla*_CTX-M_) genes in *Escherichia coli* isolated from chicken meat, chevon meat, raw milk, and human urine and stool samples collected from tribal districts of Chhattisgarh, *viz*., Jagdalpur, Dantewada, Kondagaon, and Kanker.

**Materials and Methods:**

A total of 330 samples, comprising 98 chicken meat, 82 chevon meat, 90 raw milk, and 60 human urine and stool samples, were processed for isolation of *E. coli*. Isolates were confirmed biochemically and further tested against commonly used antibiotics to know their resistant pattern. The resistant isolates were tested for ESBL production by phenotypic method followed by characterization with molecular method using multiplex-polymerase chain reaction technique.

**Results:**

Overall 57.87% (191/330) samples were found positive for *E. coli*, which include 66.32% (65/98) chicken meat, 46.34% (38/82) chevon meat, 81.11% (73/90) raw milk, and 25% (15/60) human urine and stool samples. Isolates showed the highest resistance against cefotaxime (41.36%) followed by oxytetracycline (34.03%), ampicillin (29.31%), cephalexin (24.60%), cefixime (16.75%), and ceftazidime (13.08%). Phenotypic method detected 10.99% (21/191) isolates as presumptive ESBL producers, however, molecular method detected 3.66% (7/191), 2.09% (4/191), and 0.00% (0/191) prevalence of *bla*_TEM_, *bla*_CTX-M_, and *bla*_SHV_, respectively.

**Conclusion:**

The present study indicates a high prevalence of *E. coli* in raw chicken meat, chevon meat, and milk due to poor hygienic practices. The antibiotic susceptibility test detected the presence of the resistance pattern against ESBL in *E. coli* isolated from raw chicken meat, chevon meat, milk, and also in human clinical samples is of great concern. The appearance of *E. coli* in the human food chain is alarming and requires adaptation of hygienic practices and stipulate use of antibiotics.

## Introduction

Food-borne diseases remain a major public health problem across the globe. Moreover, the situation worsens in developing countries like India due to difficulties in securing optimal hygienic food handling practices. Most of the bacterial pathogens associated with human enteric illness originate from animals and can be transmitted directly from animals to humans or indirectly through foods of animal origin, contaminated water, etc. [1,2]. Among the genus Enterobacter, *Escherichia coli* is mainly responsible for causing diarrhea, hemolytic uremic syndrome, and hemorrhagic colitis (HC) [3]. *E. coli* can contaminate the foods of animal origin, *viz*., raw milk, meat, and their products and contribute to human food-borne diseases and food spoilage. *E. coli* also causes nosocomial infections especially meningitis in infants. More than 85% of urinary tract infections are attributed to this organism [4]. Several strains of *E. coli* are recognized as important pathogens responsible for causing severe human diseases such as HC and hemolytic uremic syndrome [5].

Nowadays, curing bacterial infections in human and veterinary medicine is facing several problems due to increased antimicrobial resistance (AMR) in bacteria against the most of the antibacterial agents. The intensive use and, particularly, the misuse of antibiotics have led to the development and selection of resistant bacteria in different settings. One of the most important AMR mechanisms in Enterobacteriaceae family is production of extended-spectrum β-lactamase (ESBL) enzymes. According to Ambler classification, ESBL is divided into four main groups from A to D [6]. ESBL enzymes TEM, SHV, and CTX-M from Group A have been widely reported to be produced by *E. coli*. These enzymes can hydrolyze ampicillin, carbenicillin, oxacillin, and an extended spectrum of cephalosporins such as ceftazidime and cefotaxime [7].

The emergence of ESBL producing *E. coli* in the food-producing animals and in foods of animal origin is a growing problem worldwide [8]. There are scanty reports animals and on the association of ESBL producing enteric bacteria in humans and foods of animal origin in India as in the state of Chhattisgarh. Therefore, this study was conducted to investigate the occurrence of ESBL-producing *E. coli* in foods of animal origin and human clinical samples from different districts of Chhattisgarh, India.

## Materials and Methods

### Ethical approval

Live animals were not used in this study, so ethical approval was not necessary. Meat samples were collected from retail meat shops, and human clinical samples were collected from hospitals, private clinics, and diagnostic centers.

### Sample collection

A total of 330 samples comprising of chicken meat, chevon meat, raw milk, and human urine and stool were collected randomly during August 2014 to July 2015 from Jagdalpur, Dantewada, Kanker, and Kondagaon districts of Chhattisgarh, India. The chicken meat (n=98) and chevon meat (n=82) samples were aseptically collected from retail meat shops and slaughter houses as per the guidelines of the International Commission on Microbiological Specifications for Foods [9]. Raw milk samples (n=90) were aseptically collected from hotels/restaurants using sterile milk sample bottles. All milk samples were stored at 4°C and were cultured within 5 h. Human urine (n=56) and stool samples (n=4) were collected from hospitals, private clinics, and diagnostic centers. Human patients suffering from urinary tract infection were given dry test tube and requested to provide 10-20 ml urine sample for examination. The human stool samples were collected using sterilized dry absorbent cotton swab following the protocol of Cheesbrough [10].

### Isolation and biochemical characterization

Isolation was carried out as per method described by Agarwal *et al*. [11]. Briefly, 10 g of raw chicken and chevon meat, 1 ml of raw milk, 1 g of human stool, and 1 ml of human urine samples were taken and inoculated in sterilized MacConkey’s lactose broth (HiMedia, India) and incubated at 37°C for 24 h. Thereafter, a loop-full culture from enrichment broth was taken and streaked onto MacConkey’s lactose agar (HiMedia, India) and incubated at 37°C for 24 h. The suspected *E. coli* colonies, pink to red were picked up and further streaked on eosin methylene blue agar (HiMedia, India) and incubated at 37°C for 24 h. Dark-centered and flat colonies with metallic sheen were considered as *E. coli*. All the *E. coli* isolates were further confirmed by indole, methyl red, Voges–Proskauer, and citrate utilization biochemical tests.

### Antimicrobial susceptibility test and multiple antibiotic resistance (MAR) index

All biochemically confirmed *E. coli* isolates were tested for their antimicrobial drug susceptibility pattern on Mueller-Hinton agar (MHA) (HiMedia, India) by the disc diffusion method [12]. The antibiotics used were oxytetracycline (30 μg), cephalexin (30 μg), ciprofloxacin (5 μg), gentamicin (30 μg), cefotaxime (10 μg), ampicillin (10 μg), ceftazidime (30 μg), aztreonam (30 μg), imipenem (10 μg), cefixime (5 μg), and meropenem (10 μg) (HiMedia, India). The diameter of the zones of complete inhibition was measured and compared with the zone size interpretation chart and was graded as sensitive, intermediate, and resistant. The MAR Index was also calculated for all *E. coli* isolates, by applying formula a/b where “a” is the number of antibiotics to which an isolate was resistant and “b” is the number of antibiotics to which the isolates exposed [13].

### Phenotypic detection of ESBL producers

As per CLSI protocol, *E. coli* isolates with a zone of inhibition of ≤17 mm for aztreonam and ceftazidime, and ≤22 mm for cefotaxime in disc diffusion susceptibility testing were selected for detection of ESBLs production. For this purpose, four antibiotics cefotaxime (10 μg), ceftazidime (30 μg), aztreonam (30 μg), and cefotaxime+clavulanic acid (30+10 μg) (HiMedia, India) were used. Discs were placed on the inoculated MHA plates at a distance of 25 mm apart and incubated overnight. The *E. coli* isolates resistant to either of the cephalosporin discs and sensitive to their respective cephalosporin+clavulanic acid discs with diameter of more than 5 mm were considered as presumptive ESBL producers [13].

### Molecular characterization of ESBL-encoding genes

All the presumptive ESBL producing *E. coli* isolates were screened for the detection of *bla*_TEM_, *bla*_SHV_, and *bla*_CTX-M_ genes by multiplex-polymerase chain reaction (m-PCR) as described by Monstein *et al*. [14] with some modifications. Template DNA incorporated in PCR reactions was prepared by boiling and snap chill method as outlined by Nagappa *et al*. [15]. Purity and concentration of DNA were detected by 0.8% agarose gel electrophoresis and stored at −20°C. The recommended oligonucleotide primers specific for the *bla*_SHV_ [16], *bla*_TEM_ [14], and *bla*_CTX-M_ [17] genes used in the m-PCR assay and expected amplicon sizes are given in Table-1. All the primers used in the present study were procured from the Imperial Life Sciences (P) Limited, Gurgaon, Haryana, India. PCR reactions were performed in a total volume of 25 μl containing ×10 PCR buffer (Tris with 15 mM MgCl_2_), 25 μM of each deoxyribonucleotide triphosphate, 10 pmol of each gene-specific primers, 1U Taq polymerase, and 3 μl of template DNA. The m-PCR was done using thermocycler (Mastercycler, Eppendorf, Germany), and cycles were performed with initial denaturation of 95°C for 10 min; 30 cycles of denaturation at 94°C for 30 seconds, annealing at 60°C for 30 s, extension at 72°C for 2 min followed by a final extension step at 72°C for 10 min. After the completion of reaction cycles, the amplified products were electrophoresed on 1.5% agarose gel and stained with 0.5 μg/ml ethidium bromide. The images of ethidium bromide stained DNA bands were analyzed under UV transilluminator (Biometra) and recorded using a Gel Documentation System (Gel Doc™XR, Biorad, USA).

**Table-1:** Detail of the primers used in m-PCR amplification.

Target gene	Primer sequence	Amplicon size (bp)
*bla*_SHV_	ATG CGT TAT ATT CGC CTG TG TGC TTT GTT ATT CGG GCC AA	747
*bla*_TEM_	TCG CCG CAT ACA CTA TTC TCA GAA TGA ACG CTC ACC GGC TCC AGA TTT AT	445
*bla*_CTX-M_	ATG TGC AGY ACC AGT AAR GTK ATG GC TGG GTR AAR TAR GTS ACC AGA AYC AGC GG	593

m-PCR=Multiplex-polymerase chain reaction

## Results and Discussion

In the present study, a total of 191 (57.87%) isolates were identified as *E. coli* after morphological and biochemical characterization. The highest prevalence of *E. coli* was observed in raw milk samples followed by chicken meat, chevon meat, and human urine and stool samples (Table-2). In chicken meat samples, 66.32% prevalence of *E. coli* was recorded which is comparable with the findings of Patyal *et al*. [18], who reported 68% prevalence rate in Jaipur, Rajasthan. Similarly, Sharma and Bist [19] also reported 70% prevalence rate in chicken meat in Mathura city of Uttar Pradesh. However, lower prevalence rates of 40% were reported by Rashid *et al*. [20] in Jammu. During the present study, 46.34% chevon meat samples were found positive for *E. coli*. The level of contamination was significantly lower as compared to reports of Gangil *et al*. [21], who recorded 76% prevalence in chevon meat in Jaipur, Rajasthan, India, and on contrary, lower prevalence rates of 16.66% by Panda *et al*. [22] in Palam valley, Uttarakhand, India; 35% by Ahmad *et al*. [23] in Lahore, Pakistan; 38.09% by Dewangan [24] in Chhattisgarh, India, were reported. In milk samples, 81.1% prevalence of *E. coli* was recorded, which is lower than previously reported prevalence rates of 100% by Rai *et al*. [25] in Dehradun city, Uttarakhand, India. However, lower prevalence rates of 33.96% and 32.93% were reported by Rashid *et al*. [20] in Jammu and Kashmir, India, and Gautam *et al*. [26] in Allahabad, UP, India, respectively. The 25% prevalence of *E. coli* in human stool and urine samples was recorded in the present study, which is comparable with the findings of Rajan and Prabavathy [27], who reported 20.46% prevalence rate in urine samples in community hospital of Chennai, Tamil Nadu, India. Whereas, higher prevalence rates of 32.5% and 59% were reported by Kumar *et al*. [28] and Virpari *et al*. [29] in Anand city, Gujarat, India. Kumar *et al*. [30] in Greater Noida and Xavier *et al*. [31] in Tamil Nadu, reported lower prevalence rates of 3.88% and 15%, respectively, in human stool sample.

**Table-2:** Prevalence of *E. coli* and *bla*_TEM_, *bla*_CTX-M_, and *bla*_SHV_ gene in chicken meat, chevon meat, milk, and human clinical samples.

Types of sample	Number of samples collected	Number of positive samples (%)	ESBL production (%)			

			Phenotypic method	Molecular method		

				*bla*_TEM_	*bla*_CTXM_	*bla*_SHV_
Chicken meat	98	65 (66.32)	2 (3.07)	1 (1.53)	1 (1.53)	0
Chevon meat	82	38 (46.34)	7 (18.42)	1 (2.63)	0 (0.00)	0
Raw milk	90	73 (81.11)	9 (12.32)	4 (5.47)	2 (2.73)	0
Human urine and stool samples	60 (56+4)	15 (25)	3 (20)	1 (6.66)	1 (6.66)	0
Total	330	191 (57.87)	21 (10.99)	7 (3.66)	4 (2.09)	0 (0.00)

*E. coli=Escherichia coli,* ESBL=Extended-spectrum β-lactamases

Antimicrobial susceptibility test showed that 41.36% isolates were resistant to cefotaxime followed by oxytetracycline (34.03%), ampicillin (29.31%), cephalexin (24.60%), cefixime (16.75%), and ceftazidime (13.08%). A similar type of observations was also recorded by Rashid *et al*. [20], who reported that 40% *E. coli* isolates were resistant to cefotaxime. Among 191 *E. coli* isolates, 74 isolates were resistant for two or more than two antibiotics. The highest MAR of 0.9 was recorded in one isolate, followed by 0.81 by 2 isolates, 0.72 by 2 isolates, 0.63 by 2 isolates, 0.54 by 13 isolates, 0.45 by 10 isolates, 0.36 by 8 isolates, 0.27 by 12 isolates, and 0.18 by 24 isolates. 57 isolates were resistant to single antibiotic representing 0.09 MAR index. On contrary, 60 isolates were completely sensitive for all antibiotics. The prevalence of MAR in *E. coli* isolates was also reported by Krumperman [13] and Jaulkar *et al*. [32]. The pathogens with higher indices of MAR in foods of animal origin may possibly be introduced from the environment. The wide use and abuse of antibiotic in mass production of livestock has promoted the emergence of MAR *E. coli* in animals. MAR *E. coli* isolates were rarely found among animals, in which antibiotics are seldom or never used [13].

**Figure 1:**
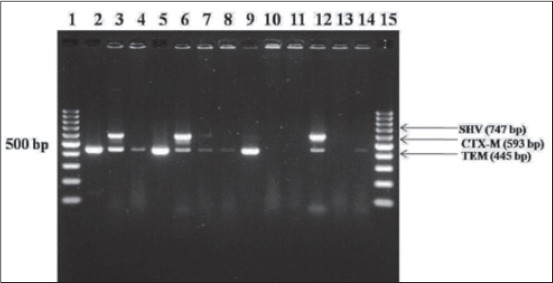
*Escherichia coli* isolates from foods of animal origin (chicken meat, chevon meat, and raw milk), human urine and stool samples showing resistance against extended-spectrum β-lactamases antibiotic by multiplex-polymerase chain reaction. Lane 1 and 15: 100 bp ladder, Lane 2 and 5: Positive control, Lane 3, 6, 7, 12: Positive for *bla*_TEM_ and *bla*_CTX-M_ genes, Lane 4, 9, 14: Positive for *bla*_TEM_ gene, Lane 10, 11, 13: Negative *E. coli* isolates.

Out of 191 *E*. *coli* isolates, 21 (10.99%) were phenotypically identified as presumptive ESBL producers. Among 21 isolates, 9 (12.32%), 7 (18.42%), 2 (3.07), and 3 (20%) isolates were from milk, chevon meat, chicken meat, and human urine and stool samples, respectively (Table-2). Rasheed *et al*. [33] reported 12.5% ESBL producer isolates by phenotypic method in chicken meat which is in accordance to the present findings. The genotypic methods help us to confirm the presence of genes in *E. coli* isolates which are responsible for ESBL production. Out of 21 *E. coli* presumptive ESBL producers, seven isolates expressed either one or two genes in m-PCR. The overall prevalence of *bla*_TEM_ and *bla*_CTX-M_ genes among *E. coli* isolates recorded was 3.66% and 2.09%, respectively. The prevalence rate of *bla*_TEM_ gene among chevon meat, chicken meat, milk, and human clinical isolates were 2.63%, 1.53%, 5.47%, and 6.66%, respectively. The prevalence rate of *bla*_CTX-M_ gene in milk, chevon meat, and clinical samples were 2.73% (2/73), 1.53% (1/65), and 6.66% (1/15), respectively (Table-2). 2 milk and 1 chevon *E. coli* isolates displayed expression of *bla*_TEM_ gene only and 2 milk, 1 chicken meat, and 1 stool isolates were harbored both *bla*_TEM_ and *bla*_CTX-M_ genes, whereas none of the isolates expressed the *bla*_SHV_ gene (Figure-1). Similarly, Apaka *et al*. [34] also reported a higher prevalence of *bla*_TEM_ gene (100%) than *bla*_CTX-M_ (37.5%) *bla*_SHV_ (4.1%) gene. Amplification of whole genomic DNA increased the possibility of detection, compared to the amplification of plasmid DNA alone, suggesting beta-lactamase expression is controlled by both chromosomal and plasmids DNA. Sometimes, multiple genes are responsible for the production of ESBL in single isolates. Thus, the m-PCR was used for detection of two or more genes simultaneously in a single isolate. This method provided an efficient, rapid differentiation of ESBL in selected species of *Enterobacteriacea*e and provided an efficient, rapid screening of large number of isolates, and could be used as a rapid tool for epidemiological studies among ESBL isolates.

## Conclusion

The overall prevalence of *E. coli* in chicken meat, chevon meat, milk, and human clinical samples was found 57.87%. The study reveals that meat and milk samples are frequently contaminated with antimicrobial resistant bacteria due to the intensive use of antimicrobial agents in animal husbandry and dairy sector. ESBL producing *E. coli* strains were also isolated chicken meat, chevon meat, milk, and human clinical samples. The m-PCR assay confirmed that 3.66% isolates were harbored the *bla*_TEM_ gene and 2.09% *bla*_CTX-M_ gene. These multidrug-resistant and ESBL producing *E. coli* strains can be transmitted to the human population after consumption of meat, milk, and their products. The appropriate antibiotic policy and infection control strategy in hospital settings are crucial to overcome the problems associated with infections by ESBL producing strains in humans. Thus, there is need for continued monitoring and surveillance of antimicrobial-resistant and ESBL producing *E. coli* at different levels, *viz*., animals, human and environment, and factors that contribute to their emergence and spread.

## Authors’ Contributions

SS designed and planned this research work. B collected the samples and executed the isolation, biochemical, molecular characterization work and carried out the antibiotic sensitivity assay of all isolates. AP analyzed the data and monitored the isolation, biochemical characterization, and antibiotic sensitivity assay. AP and NEG were involved in the molecular characterization experiment. All authors contributed equally in preparation and revision of the manuscript. All authors read and approved the final manuscript.
